# Assessing the effectiveness of camrelizumab plus apatinib versus sorafenib for the treatment of primary liver cancer: a single-center retrospective study

**DOI:** 10.1038/s41598-023-40030-x

**Published:** 2023-08-16

**Authors:** Dongbo Chen, Yichun Wang, Xiangxun Chen, Mei Kang, Liyang Zhu

**Affiliations:** Department of Radiation Oncology, The First Affiliated Hospital of Anhui Medical University, No. 120, Wanshui Road, Hefei, 230000 Anhui People’s Republic of China

**Keywords:** Cancer, Cancer therapy, Gastrointestinal cancer, Tumour immunology

## Abstract

Although the effectiveness of camrelizumab plus apatinib has been confirmed in a phase II clinical study, the efficacy of camrelizumab plus apatinib versus sorafenib for primary liver cancer (PLC) remains unverified. We retrospectively collected the data of 143 patients with PLC who received camrelizumab plus apatinib or sorafenib as the first-line treatment at The First Affiliated Hospital of Anhui Medical University from April 2018 to November 2021. Of these, 71 patients received an intravenous injection of camrelizumab 200 mg (body weight ≥ 50 kg) or 3 mg/kg (body weight < 50 kg) followed by an oral dosage of apatinib 250 mg/day every 3 weeks and 72 patients received sorafenib 400 mg orally, twice a day in 28-day cycles. The primary outcomes were overall survival and progression-free survival. The secondary outcomes were objective response rate, disease control rate, and safety. The median median progression-free survival and median overall survival with camrelizumab plus apatinib and sorafenib were 6.0 (95% confidence interval (CI) 4.2–7.8) and 3.0 months (95% CI 2.3–3.7) and 19.0 (95% CI 16.4–21.6) and 12.0 months (95% CI 8.9–15.1), respectively (death hazard ratio: 0.61, *P* = 0.023). Grade 3/4 treatment-related adverse events were noted in 50 (70.4%) patients in the camrelizumab plus apatinib group and 19 (26.4%) patients in the sorafenib group. Two treatment-related deaths were recorded. Clinically significant improvements were observed in overall survival and progression-free survival with camrelizumab plus apatinib versus sorafenib. Although the side effects of camrelizumab plus apatinib are relatively high, they can be controlled.

## Introduction

Primary liver cancer (PLC) is one of the most common cancers in the worldwide^1,2^. The common treatment methods for PLC include surgical resection, liver transplantation, and local ablation. Transcatheter arterial chemoembolization (TACE) is an effective treatment for noncurative PLC^3,4^. Sorafenib, an oral multi-kinase inhibitor, is the first anti-cancer drug that effectively improved the overall survival (OS) rate ^5,6^. The main treatment method for various types of cancer includes immunotherapy to enhance host anti-immunity by blocking programmed cell death protein 1 (PD-1)/anti-programmed death ligand 1 (PD-L1) interaction. Phase I/II trials of PD-1 inhibitor monotherapy, nivolumab or pembrolizumab, in advanced hepatocellular carcinoma (HCC) have shown clinically significant response rates (17–20%)^7,8^. However, in the subsequent phase III trial, neither of the two drugs reached the study endpoint^9,10^. Some active intrinsic immune-evasion pathways, including over-expression of vascular endothelial growth factor (VEGF), are associated with the development and progression of liver cancer. The Imbrave150 study demonstrated that compared with sorafenib, the median OS was significantly improved with atezolizumab plus bevacizumab (19.2 vs. 13.4 months)^11,12^. Based on this study, the combined treatment of atezolizumab plus bevacizumab is recommended as the first-line standard treatment. However, not all patients are candidates for atezolizumab plus bevacizumab combination therapy and it is not a cost-effective strategy for the first-line systemic treatment of unresectable PLC from the patients’ perspective^13^. Camrelizumab is a high-affinity, humanized monoclonal antibody that binds to PD-1 and has been approved for the treatment of patients with multiple cancers^14,15^. The phase II clinical study showed that the objective response rate (ORR) of camrelizumab in patients with advanced HCC who were previously treated was 14.7% and the OS probability at 6.0 months was 74.4%^16^. Apatinib is a selective vascular endothelial growth factor-2 tyrosine kinase inhibitor (TKI), with clinical efficacy proven in ovarian and gastric cancers^17,18^. The phase III trial showed that the median OS of apatinib as a second-line or later treatment for patients with advanced HCC was higher than that of patients treated with a placebo (8.7 vs. 6.8 months)^19^. Camrelizumab and apatinib alone have limited efficacy. However, a phase II clinical study showed that camrelizumab plus apatinib was a significant and effective alternative to the first-line approved treatment^20^. There are no available large sample clinical trials comparing sorafenib with camrelizumab plus apatinib in PLC. Therefore, we herein compared the efficacy of camrelizumab plus apatinib versus sorafenib and evaluated the toxicity and side effects.

## Materials and methods

### Study design and participants

**Figure 1:**
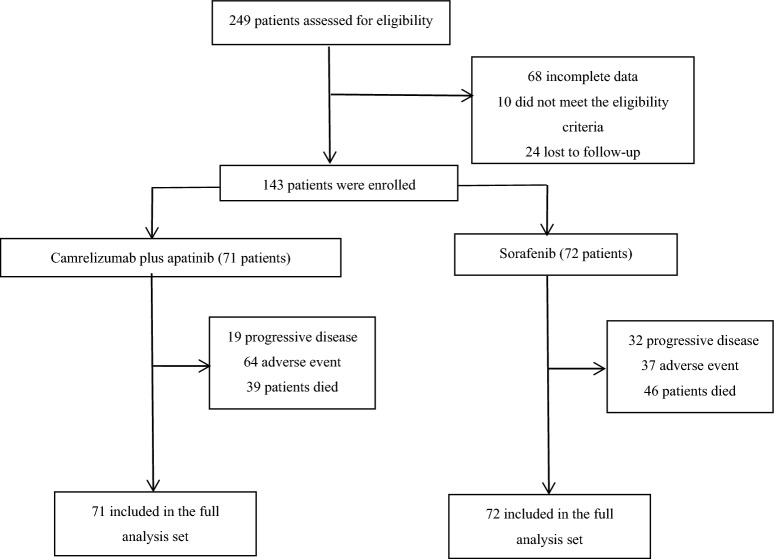
Study flow chart.

We conducted a single-center retrospective study on 143 patients with PLC who received camrelizumab plus apatinib or sorafenib as the first-line treatment at the First Affiliated Hospital of Anhui Medical University from April 2018 to November 2021 (Fig. 1). As of the data cut-off on November 30, 2022, 71 patients received camrelizumab plus apatinib and 72 received sorafenib. The baseline characteristics of patients in different treatment groups were similar (Table 1). The research was approved by the Ethical Committee of the First Affiliated Hospital of Anhui Medical University (approval number: quick-PJ 2023-04-32). Due to the retrospective design of the study, patients' written consent was waived.

**Table 1:** Baseline characteristics of the patients.

Variable	All (n = 143)	Sorafenib Group (n = 72)	Camrelizumab plus apatinib group (n = 71)	*P*-Value
Age, years	57 (28–84)	57 (39–84)	57 (28–79)	0.362
Male	125 (87.4%)	61 (84.7%)	64 (90.1%)	0.329
Histologic subtypes	47 (32.9%)	21 (29.2%)	26 (36.6%)	0.573
HCC	37 (78.8%)	17 (81.0%)	20 (76.9%)	
ICC	9 (19.1%)	4 (19.0%)	5 (19.2%)	
Mixed HCC–ICC	1 (2.1%)	0	1 (3.8%)	
ECOG PS				0.509
0	12 (8.4%)	7 (9.7%)	5 (7.0%)	
1	101 (70.6%)	53 (73.6%)	58 (81.7%)	
2	30 (21.0%)	12 (16.7%)	8 (11.3%)	
BCLC stage				0.929
A	28 (19.6%)	15 (20.8%)	13 (18.3%)	
B	30 (21.0%)	15 (20.8%)	15 (21.1%)	
C	85 (59.4%)	42 (58.3%)	43 (60.6%)	
Child-Pugh class				0.116
A	87 (60.8%)	38 (52.8%)	49 (69.0%)	
B	52 (36.4%)	31 (43.1%)	21 (29.6%)	
C	4 (2.8%)	3 (4.2%)	1 (1.4%)	
Tumor distribution				0.188
Single	45 (31.5%)	19 (26.4%)	26 (36.6%)	
Multiple	98 (69.0%)	53 (73.6%)	45 (63.4%)	
ALBI grade				0.052
1	50 (35.0%)	19 (26.4%)	31 (43.7%)	
2	82 (57.3%)	45 (62.5%)	37 (52.1%)	
3	11 (7.7%)	8 (11.1%)	3 (4.2%)	
Extrahepatic metastasis	67 (46.9%)	33 (45.8%)	34 (47.9%)	0.806
Macrovascular invasion	47 (32.9%)	22 (30.6%)	25 (35.2%)	0.553
HBV infection	99 (69.2%)	51 (70.8%)	48 (67.6%)	0.676
Cirrhosis	81 (56.6%)	45 (62.5%)	36 (50.7%)	0.155
Previous treatment				
Surgery	46 (32.2%)	20 (27.8%)	26 (36.6%)	0.258
TACE/TAE/RFA	69 (48.3%)	32 (44.4%)	37 (52.1%)	0.359
Laboratory parameters				
Hb (g/L, mean ± SD)	126.27 ± 21.18	122.19 ± 22.61	130.46 ± 18.86	0.019
Platelet (10^9^/L, mean ± SD)	137.42 ± 71.01	129 ± 75.56	145.14 ± 65.66	0.202
WBC (10^12^/L, mean ± SD)	5.60 ± 2.76	5.33 ± 2.77	5.87 ± 2.74	0.241
Neutrophils (10^9^/L, mean ± SD)	3.89 ± 2.39	3.77 ± 2.53	4.00 ± 2.25	0.564
LMR (median, mean ± SD)	3.72 ± 3.49	3.22 ± 1.74	4.24 ± 4.59	0.090
ALT (U/L, mean ± SD)	55.59 ± 122.54	59.03 ± 158.43	52.06 ± 69.52	0.734
AST (U/L, mean ± SD)	86.88 ± 188.54	104.18 ± 256.44	69.09 ± 65.88	0.264
TBIL (mmol/L, mean ± SD)	31.84 ± 55.92	22.18 ± 16.75	41.81 ± 76.24	0.041
ALP (U/L, mean ± SD)	188.87 ± 147.57	202.23 ± 146.19	175.32 ± 148.79	0.284
ALB (g/L, mean ± SD)	36.69 ± 5.96	35.71 ± 5.98	37.70 ± 5.82	0.782
GGT (U/L, mean ± SD)	190.21 ± 219.10	162.26 ± 172.04	218.57 ± 256.45	0.083
AFP (ng/ml, mean ± SD)	8516.70 ± 44,763.78	12,670.13 ± 61,550.50	4480.27 ± 16,285.36	0.288

Values are presented as median (interquartile range) or number (%).

*AFP* Alpha-fetoprotein, *ALB* Albumin, *ALBI* Albumin-bilirubin, *ALP* Alkaline phosphatase, *ALT* Alanine aminotransferase, *AST* Aspartate aminotransferase, *BCLC* Barcelona clinic liver cancer, *ECOG PS* Eastern Cooperative Oncology Group performance status, *GGT* Gamma-glutamyl transferase, *Hb* Hemoglobin, *HBV* Hepatitis B virus, *LMR* Lymphocyte-to-monocyte ratio, *TACE* Transcatheter arterial chemoembolization, *TAE* Transarterial embolization, *RFA* Radiofrequency ablation, *TBIL* Total bilirubin, *WBC* White blood cell.

### Study inclusion criteria

The inclusion criteria were as follows: (1) Patients aged > 18 years old; (2) those with a histological or radiological diagnosis as PLC according to the American Association for the Study of Liver Diseases criteria^21^; (3) those who never received any prior systematic treatment for PLC, had an Eastern Cooperative Oncology Group score of ≤ 2, and classified as Barcelona Clinic Liver Cancer (BCLC) stage A–C; (4) those with at least one measurable lesion according to Response Evaluation Criteria in Solid Tumors (RECIST) v1.1 standard; and (5) those who completed the follow-up until death or study termination. The exclusion criteria were as follows: (1) patients who received prior systemic treatment; (2) pregnant and lactating women; (3) those with cognitive dysfunction; (4) those who had other tumors in the past (within 5 years) or at the same time; (5) those with cardiopulmonary insufficiency; (6) those with abnormal coagulation function and bleeding tendency; and (7) those with uncontrollable hypertension.

### Treatment administration and outcome measures

Of the 143 patients, 71 received an intravenous injection of camrelizumab 200 mg (body weight ≥ 50 kg) or 3 mg/kg (body weight < 50 kg) (Jiangsu Hengrui Medicine Co., Ltd., Jiangsu, China) followed by an oral dosage of apatinib 250 mg/day (Jiangsu Hengrui Medicine Co., Ltd., Jiangsu, China) every 3 weeks and 72 patients received sorafenib 400 mg orally (Bayer, Leverkusen, Germany), twice a day in 28-day cycles.

The investigator would not give treatment until the clinical benefits were lost after a comprehensive evaluation of radiological and biochemical data and clinical status (such as symptom deterioration, pain secondary to disease, or unacceptable toxicity). The primary outcomes were OS and progression-free survival (PFS) based on the RECIST v1.1 standard. The secondary outcomes were ORR, disease control rate (DCR), and safety. The tumor was evaluated using computed tomography or magnetic resonance imaging at baseline, every 6–9 weeks. To continuously evaluate the safety, the patient's vital signs and clinical laboratory test results were recorded and the incidence and severity of adverse events (AEs) were evaluated according to the National Cancer Institute Common Terminology Criteria for Adverse Events Version 4.0.

### Statistical analysis

The Kaplan–Meier analysis was performed using SPSS 27.0 (IBM Corp., Armonk, NY, USA) and R version 4.2.2 (R Foundation for Statistical Computing, Vienna, Austria). All data were represented by mean ± standard deviation and n (%). Student's t-test (or Mann–Whitney test) was used for the comparison of continuous variables and the chi-square test or Fisher exact test for categorical variables. The Kaplan–Meier method was used to estimate OS and PFS. Multivariate Cox regression analysis was performed to determine independent prognostic factors.

## Results

### Baseline characteristics

The data of 143 patients with PLC patients who received camrelizumab plus apatinib (71 patients) or sorafenib (72 patients) as the first-line treatment at The First Affiliated Hospital of Anhui Medical University from April 2018 to November 2021 were retrospectively collected. As of the data cut-off on November 30, 2022, the median follow-up time was 15.0 months. Among the patients, 39 (54.9%) died in the camrelizumab plus apatinib group and 46 (63.9%) in the sorafenib group. The median age of the entire cohort was 57 (range: 28–84) years, and the majority were men (87.4%). A total of 47 patients underwent pathological diagnosis, including 37 cases of HCC, 9 cases of intrahepatic cholangiocellular carcinoma, and 1 case of mixed cell carcinoma.

Hepatitis B virus is the main cause of PLC, accounting for 69.2% (99/143) of all cases. According to the BCLC staging system, 59.4% (85/143) of patients were classified as BCLC stage C. In terms of liver function, 60.8% (87/143) and 36.4% (52/143) of patients were classified as Child-Pugh A or B, respectively. Vascular invasion and extrahepatic metastasis were noted in 32.9% (47/143) and 46.9% (67/143) patients, respectively. Among the patients, 46 (32.2%) underwent surgery before receiving first-line systemic therapy and 69 (48.3%) underwent transcatheter arterial chemoembolization/transarterial embolization/radiofrequency ablation.

### Efficacy

Table 2 shows the treatment response during treatment. According to the RECIST standard 1.1, complete response was achieved in one patient (1.4%) in the sorafenib group and one (1.4%) in the camrelizumab plus apatinib group. The ORR (22.5% vs.11.1%) and DCR (73.2% vs. 51.4%, *P* = 0.013) of the camrelizumab plus apatinib group were higher than those of the sorafenib group.

**Table 2:** Clinical response of patients during treatment.

Response	Sorafenib (n = 72)	Camrelizumab plus apatinib (n = 71)
Complete response	1 (1.4%)	1 (1.4%)
Partial response	7 (9.7%)	15 (21.1%)
Stable disease	29 (40.3%)	36 (50.7%)
Progressive disease	32 (44.4%)	19 (26.8%)
Not evaluabe	1 (1.4%)	0
Objective response rate	8 (11.1%)	16 (22.5%)
Disease control rate	37 (51.4%)	52 (73.2%)

**Figure 2:**
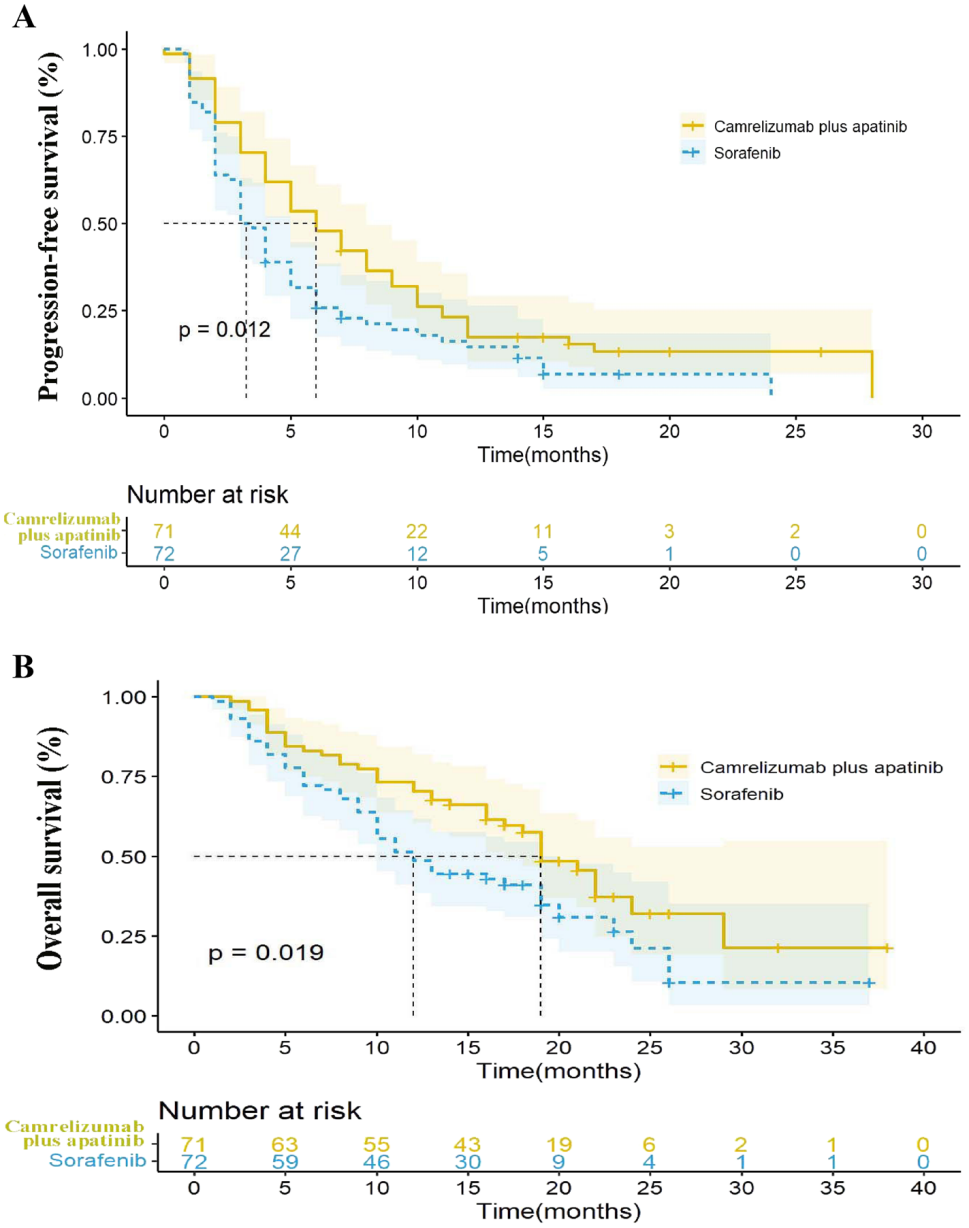
Kaplan–Meier analysis of survival outcomes in patients treated with camrelizumab plus apatinib versus sorafenib. (**A**) Progression-free survival; (**B**) Overall survival.

The median OS of the two groups was 19.0 months (95% confidence interval (CI): 16.4–21.6) and 12.0 months (95% CI 8.9–15.1), respectively, indicating a 39% reduction in the risk of death (95% CI 0.399–0.935, *P* = 0.023, hazard ratio (HR) = 0.610) (Fig. 2). The 12-month and 18-month survival rates of the camrelizumab plus apatinib group were 70.4% (50/71) and 57.7% (41/71), respectively whereas those of the sorafenib group were 48.6% (35/72) and 34.7% (25/72), respectively. The median PFS of the camrelizumab plus apatinib group was 6.0 months (95% CI 4.2–7.8) and that of sorafenib was 3.0 months (95% CI 2.3–3.7) with an HR of 0.65 (*P* = 0.012) (Fig. 2). The camrelizumab plus apatinib groups showed better clinical benefits than the sorafenib group. Cox multivariate analysis confirmed that the BCLC stage (HR = 0.547, 95% CI 0.329–0.910, *P* = 0.020) was an independent prognostic factor for OS (Table 3).

**Table 3:**
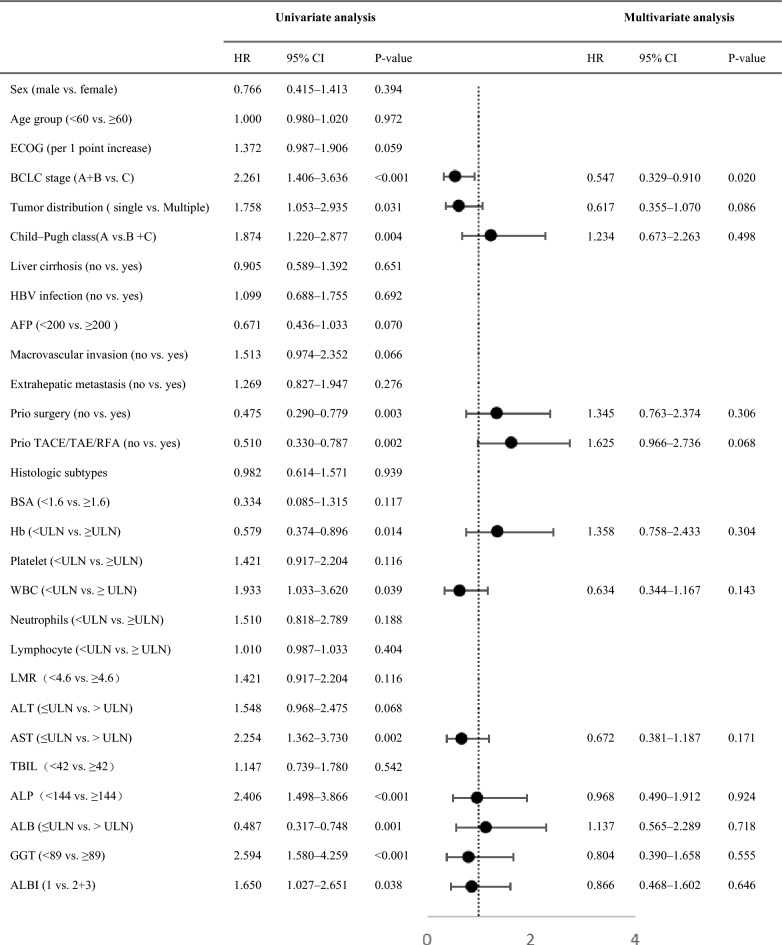
Univariate Cox proportional hazards regression model analysis for OS.

*AFP* Alpha-fetoprotein, *ALB* Albumin, *ALBI* Albumin-bilirubin, *ALP* Alkaline phosphatase, *ALT* Alanine aminotransferase, *AST* Aspartate aminotransferase, *BCLC* Barcelona clinic liver cancer, *BSA* Body surface area, *CI* Confidence interval, *ECOG PS* Eastern Cooperative Oncology Group performance status, *GGT* Gamma-glutamyl transferase, *Hb* Hemoglobin, *HBV* Hepatitis B virus, *HR* Hazard ratio, *LMR* Lymphocyte-to-monocyte ratio, *TACE* Transcatheter arterial chemoembolization, *TAE* Transarterial embolization, *OS* Overall survival, *RFA* Radiofrequency ablation, *TBIL* Total bilirubin, *WBC* White blood cell.

### Safety

AEs were reported by 90.1% (64/71) of patients in the camrelizumab plus apatinib group and 51.4% (37/72) in the sorafenib group (Table 4). Moreover, 18.3% (13/71) of patients in the camrelizumab plus apatinib group and 11.1% (8/72) of patients in the sorafenib group discontinued treatment due to AEs. The most common treatment-related AEs in the camrelizumab plus apatinib group were hypertension in 48 (67.6%) patients, thrombocytopenia in 40 (56.3%) patients, and elevated serum aspartate aminotransferase level in 39 (54.9%) patients. In the sorafenib group, the most common treatment-related AEs were hand-foot syndrome in 22 (30.6%) patients and elevated serum aspartate aminotransferase levels in 21 (29.2%) patients. Grade 3/4 AEs occurred in 50 (70.4%) patients of the camrelizumab plus apatinib group and 19 (26.4%) of the sorafenib group. The most common grade 3/4 AEs in the camrelizumab plus apatinib group were hypertension (33.8%, n = 24), elevated serum aspartate aminotransferase level (12.7%, n = 9), and thrombocytopenia (9.7%, n = 7). There was a higher rate of hypertension (33.8% vs. 12.5%, p = 0.003) and elevated serum aspartate aminotransferase level (12.7% vs. 2.8%, *P* = 0.026) as grade 3/4 AEs in the camrelizumab plus apatinib group than in the sorafenib group. In the camrelizumab plus apatinib group, four patients developed upper gastrointestinal bleeding, two discontinued treatment, and one had drug-related death due to upper gastrointestinal bleeding and liver lesions entering pleural cavity rupture. Bleeding events that occur during or after progression may be assessed as those related to progression. One patient in the sorafenib group died of unknown causes after1 month of medication.

**Table 4:** Adverse events.

Any	Sorafenib group (n = 72)		Camrelizumab plus apatinib group (n = 71)	
	37 (51.4%)		64 (90.1%)	
Grade 3/4	19 (26.4%)		50 (70.4%)	
Led to treatment discontinuation	8 (11.1%)		13 (18.3%)	
Led to death	1 (1.4%)		1 (1.4%)	
	Grade	Grade	Grade	Grade
	Any	3/4	Any	3/4
Hypertension	15 (20.8%)	9 (12.5%)	48 (67.6%)	24 (33.8%)
Proteinuria	11 (15.3%)	1 (1.4%)	34 (47.9%)	1 (1.4%)
Hand-foot syndrome	22 (30.6%)	7 (9.7%)	25 (35.2%)	6 (8.5%)
Reactive cutaneous capillary endothelial proliferation	0	0	18 (25.4%)	0
Hematuria	14 (19.4%)	0	14 (19.7%)	0
Abdominal pain	12 (16.7%)	0	22 (31.0%)	1 (1.4%)
Abdominal distention	10 (13.9%)	0	18 (25.4%)	0
Leukopenia	5 (6.9%)	0	32 (45.1%)	4 (5.6%)
Anaemia	10 (13.9%)	2 (2.8%)	38 (53.5%)	2 (2.8%)
Neutropenia	5 (6.9%)	1 (1.4%)	28 (39.4%)	6 (8.5%)
Thrombocytopenia	10 (13.9%)	0	40 (56.3%)	7 (9.9%)
Aspartate aminotransferase increased	21 (29.2%)	2 (2.8%)	39 (54.9%)	9 (12.7%)
Alanine aminotransferase increased	14 (19.4%)	2 (2.8%)	34 (47.9%)	5 (7.0%)
Hyperbilirubinemia	10 (13.9%)	0	34 (47.9%)	6 (8.5%)
Hypoalbuminemia	10 (13.9%)	1 (1.4%)	31 (43.7%)	0
Hypokalemia	4 (5.6%)	1 (1.4%)	7 (9.9%)	2 (2.8%)
Hypothyroidism	7 (9.7%)	0	9 (12.7%)	0
Upper gastrointestinal haemorrhage	1 (1.4%)	1 (1.4%)	2 (2.8%)	2 (2.8%)

## Discussion

Targeted combined immunotherapy can improve the viewpoint for the treatment of liver cancer and provides a new treatment standard. Moreover, it has an improved long-term efficacy compared with that of other unresectable liver cancer treatments^22–24^. Atezolizumab plus bevacizumab and camrelizumab plus apatinib are the currently available first-line treatments for advanced PLC. There are disagreements about which scheme should be selected as the initial treatment method for patients with newly diagnosed PLC. In a phase II clinical study of camrelizumab plus apatinib including 70 first-line and 120 s-line patients, the ORR of the first-line patients was 34.3% and that of the second-line patients was 22.5%. The median PFS of the two groups was 5.7 and 5.5 months, respectively. The 12-month survival rates were 74.7% and 68.2% respectively, and the median OS was not reached^20^. The United States Food and Drug Administration approved the international multi-center phase III clinical trial of first-line treatment of HCC with camrelizumab plus apatinib to be simultaneously conducted in the United States, Europe, and China, and the experimental results are to be published. Yang et al. enrolled 83 patients and retrospectively analyzed the clinical efficacy of camrelizumab plus apatinib versus sorafenib as the first-line treatment of HCC. The results showed that the median PFS and OS of the camrelizumab plus apatinib group were 8.1 and 13.3 months, respectively, which were significantly higher than those of the sorafenib group (PFS = 5.3, OS = 9.2)^25^.In the present study, we evaluated the efficacy and toxicity of camrelizumab plus apatinib in the real world in Chinese patients with PLC. In this study, the ORR and DCR of 71 patients in the camrelizumab plus apatinib group (22.5% and 73.2%, respectively) were higher than those of the sorafenib group (11.1% and 51.4%, respectively). The median OS and PFS of the camrelizumab plus apatinib group were 19.0 and 6.0 months, respectively, which was also significantly higher than the median OS (12.0 months) and median PFS (3.0 months) of the sorafenib group. The therapeutic effect of this study was slightly lower than that of the phase II clinical trial. Considering that the group of patients enrolled in our study was more diverse than that of the phase II clinical trial, including those with Child–Pugh Class C, Eastern Cooperative Oncology Group 2, abnormal laboratory indicators and elderly patients. This suggests that camrelizumab plus apatinib can be safely used, even exceeding the strict inclusion criteria of phase II clinical research. Moreover, COX multivariate analysis confirmed that the BCLC stage was an independent prognostic factor for OS.

In the phase III trial of a single drug, apatinib, as a second-line or late treatment for patients with advanced HCC, treatment-related adverse events (TRAEs) occurred in 97% of patients, and grade 3/4 TRAEs were observed in 77% of patients. The most common grade 3/4 TRAEs were hypertension (28%), hand-foot syndrome (18%), and decreased platelet count (13%)^19^. The phase II clinical trial of a single drug, camrelizumab, used in patients with advanced HCC who had received prior treatment showed that the most common TRAEs at any level were reactive cutaneous capillary endothelial proliferation (67%), elevated serum aspartate aminotransferase level (24%), and proteinuria (23%). Grade 3**/**4 TRAEs accounted for 22%, with the most common TRAEs being increased elevated serum aspartate aminotransferase level (5%) and decreased neutrophil count (3%)^16^. The phase II trial of camrelizumab plus apatinib in the treatment of advanced HCC showed that 99.5% of patients had at least one TRAE. Hypertension (72.6%), elevated serum aspartate aminotransferase level (63.2%), proteinuria (61.6%), and hyperbilirubinemia (61.6%) were the most common TRAEs at any level. Patients with grade ≥ 3 TRAEs accounted for 77.4%, with the most common TRAEs being hypertension (34.2%). Severe TRAEs were noted in 28.9% of patients. Treatment-related death occurred in 1.1% of patients^20^. In the present study, the AEs of patients in the camrelizumab plus apatinib group were consistent with those in phase II clinical trials and higher than those in phase III clinical trials of atezolizumab plus bevacizumab^26^.Grade 3**/**4 AEs occurred in 70.4% of the camrelizumab plus apatinib group and 26.4% of the sorafenib group. The most common grade 3/4 AEs in the camrelizumab plus apatinib group were hypertension, elevated serum aspartate aminotransferase level, and thrombocytopenia. The incidence of grade 3 and above AEs in the camrelizumab plus apatinib group was higher than that in the sorafenib, camrelizumab alone, and apatinib alone groups, but most of the AEs in the camrelizumab plus apatinib group could be controlled by drug treatment. In the camrelizumab plus apatinib group, four patients developed upper gastrointestinal bleeding, two patients discontinued treatment, and one patient had drug-related death due to upper gastrointestinal bleeding and liver lesions entering pleural cavity rupture. Variceal bleeding is one of the main causes of death in patients with liver cirrhosis and PLC; however, in a meta-analysis of 27 randomized trials, the total incidence of all-grade and high-grade bleeding events in patients treated with anti-angiogenic TKI was 9.1% and 1.3%, respectively. The risk ratio of all levels of bleeding was higher in patients receiving TKI treatment than that in the controls^27–29^. The mechanism of TKI bleeding remains complex and has not been clarified. Considering that apatinib increases the risk of upper gastrointestinal bleeding, it should be evaluated for patients with a high risk of bleeding using oesophagogastroduodenoscopy before starting the combined treatment of camrelizumab plus apatinib^19,30^.

Nonetheless, our study has some limitations. First, retrospective data cannot replace first-level evidence from prospective studies. Due to the small sample size, we did not further analyze the differences in OS between patients who met the conditions of phase II clinical trials and those who did not. Second, the real-life background of the study lead to a lack of standardization in clinical practice, and our results should be considered speculative, particularly regarding the identification of prognostic factors.

In conclusion, we confirmed that camrelizumab plus apatinib has better clinical benefits than sorafenib and controllable adverse effects. Therefore, camrelizumab plus apatinib will be a significant and effective first-line treatment option.
